# Galactosylated Chitosan Oligosaccharide Nanoparticles for Hepatocellular Carcinoma Cell-Targeted Delivery of Adenosine Triphosphate

**DOI:** 10.3390/ijms140815755

**Published:** 2013-07-29

**Authors:** Xiu Liang Zhu, Yong Zhong Du, Ri Sheng Yu, Ping Liu, Dan Shi, Ying Chen, Ying Wang, Fang Fang Huang

**Affiliations:** 1 Department of Radiology, the Second Affiliated Hospital, Zhejiang University School of Medicine, 88 Jiefang Road, Hangzhou 310009, China; E-Mails: zhuxiul@yeah.net (X.L.Z.); danshi2012@163.com (D.S.); dchenying2000@163.com (Y.C.); wyyw2000@yeah.net (Y.W.); ffhuanghz@126.com (F.F.H.); 2 Department of Pharmaceutics, College of Pharmaceutical Sciences, Zhejiang University, 866 Yuhangtang Road, Hangzhou 310058, China; E-Mails: duyongzhong01@126.com (Y.Z.D.); liuping1988@126.com (P.L.)

**Keywords:** galactosylated chitosan oligosaccharide, adenosine triphosphate, nanoparticles, hepatocyte uptake, targeted drug delivery

## Abstract

Nanoparticles composed of galactosylated chitosan oligosaccharide (Gal-CSO) and adenosine triphosphate (ATP) were prepared for hepatocellular carcinoma cell-specific uptake, and the characteristics of Gal-CSO/ATP nanoparticles were evaluated. CSO/ATP nanoparticles were prepared as a control. The average diameter and zeta potential of Gal-CSO/ATP nanoparticles were 51.03 ± 3.26 nm and 30.50 ± 1.25 mV, respectively, suggesting suitable properties for a drug delivery system. Subsequently, the cytotoxicity of Gal-CSO/ATP nanoparticles were examined by the methyl tetrazolium (MTT) assay, and the half maximal inhibitory concentration (IC_50_) values were calculated with HepG2 (human hepatocellular carcinoma cell line) cells. The results showed that the cytotoxic effect of nanoparticles on HepG2 cells was low. In the meantime, it was also found that the Gal-CSO/ATP nanoparticles could be uptaken by HepG2 cells, due to expression of the asialoglycoprotein receptor (ASGP-R) on their surfaces. The presented results indicate that the Gal-CSO nanoparticles might be very attractive to be used as an intracellular drug delivery carrier for hepatocellular carcinoma cell targeting, thus warranting further *in vivo* or clinical investigations.

## 1. Introduction

Due to the recent advances in material science and nanotechnology in the past few years, nanoparticles have become very attractive for their applications in the fields of biology, medicine and bioimaging [1–5]. Nanoparticles can be used to reliably and safely deliver proteins, drugs, vaccines and other biological macromolecules into specific cells or tissues [6–8].

Chitosan is a modified natural carbohydrate polymer prepared by the partial *N*-deacetylation of the crustacean-derived natural biopolymer, chitin, and it has been proposed as an alternative, biocompatible cationic polymers that are suitable for mucosal drug and vaccine delivery [9–13] and gene delivery [14–21]. Chemically modified chitosans have great utility in controlled release and targeting studies of almost all classes of bioactive molecules.

Most chitosans are only soluble in aqueous acidic solutions below pH 6.5, where primary amino groups of chitosan are protonated. In this study, to improve water solubility of chitosan, water-soluble chitosan with lower molecular weight, chitosan oligosaccharide (CSO), was used and coupled with lactobionic acid (LA) bearing a galactose group as the specific ligand to the asialoglycoprotein receptor (ASGP-R) of hepatocellular carcinoma cells. ASGP-R are frequently used as a target, due to the high expression on the surface of hepatocytes and in hepatocellular carcinoma cell lines [22]. The binding of the galactose ligand with ASGP-R induces liver-targeted transfer [23].

Adenosine triphosphate (ATP) contains a hydrophobic adenine group, along with four negative charges arising from the attached phosphate groups. It is a crucial molecule that strongly participates in the biological activities of living creatures, usually transferring chemical energy via the formation and cleavage of the phosphoanhydride bonds catalyzed by specific enzymes [24]. Hepatic ATP levels, in particular, accurately reflect the extent of hepatic disease [25,26]. Phosphorus-31 magnetic resonance spectroscopy (31P MRS) has been utilized to assess energy states in living systems [27]. This technique permits simultaneous detection and quantitation of several cytosolic phosphorus-containing compounds involved in energy metabolism (ATP and inorganic phosphate) and membrane phospholipid metabolism (phosphomonoesters and phosphodiesters) [28]. Thus, targeting delivery of ATP to hepatopathy tissue may be an effective technology for the diagnosis of early hepatic disease by 31P MRS.

The physical characteristics of the galactosylated chitosan oligosaccharide (Gal-CSO)/ATP nanoparticles were analyzed. *In vitro* drug cumulative release rate in Phosphate Buffered Saline (PBS) (pH 7.4) and cytotoxicity studies on the HepG2 cell line were also conducted. In the current work, Gal-CSO/ATP nanoparticles were synthesized, and their application as a potential drug delivery system for targeting hepatocellular carcinoma cell was investigated. In the meantime, as a control, the CSO/ATP nanoparticles were prepared in the same preparation condition.

## 2. Results and Discussion

### 2.1. Preparation of Nanoparticles

**Figure 1:**
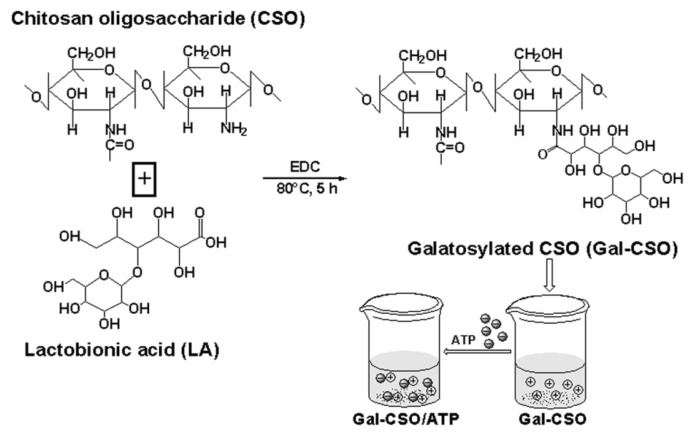
Synthetic route of galactosylated chitosan oligosaccharide (Gal-CSO) and schematic representation depicting the formation of Gal-CSO/adenosine triphosphate (ATP).

The preparation procedures of nanoparticles are illustrated in Figure 1. Gal-CSO was prepared by conjugation of LA to CSO using 1-ethyl-3-(3-dimethyl aminopropyl) carbodiimide (EDC)-mediated condensation. The LA, bearing a galactose group, was chosen, because it is known as a specific adhesive ligand to the ASGP-R of hepatocellular carcinoma cells, and its biocompatibility has already been proven [29–35].

### 2.2. Characteristics of Nanoparticles

#### 2.2.1. Morphology, Particle Size, Zeta Potential and Stability

**Figure 2:**
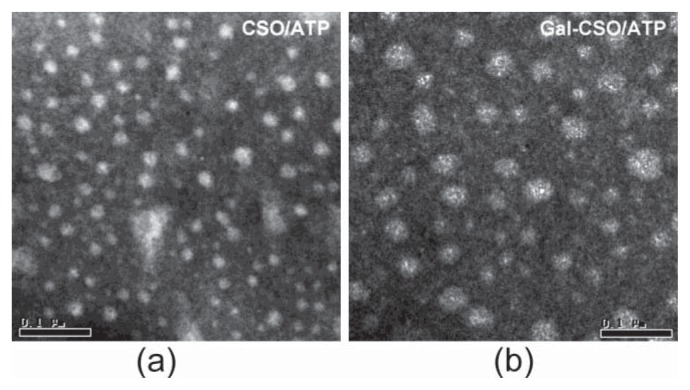
Representative TEM images of (**a**) CSO/ATP and (**b**) Gal-CSO/ATP nanoparticles. Note: the bar is 0.1 μm. please check and provide clearer figures.

**Figure 3:**
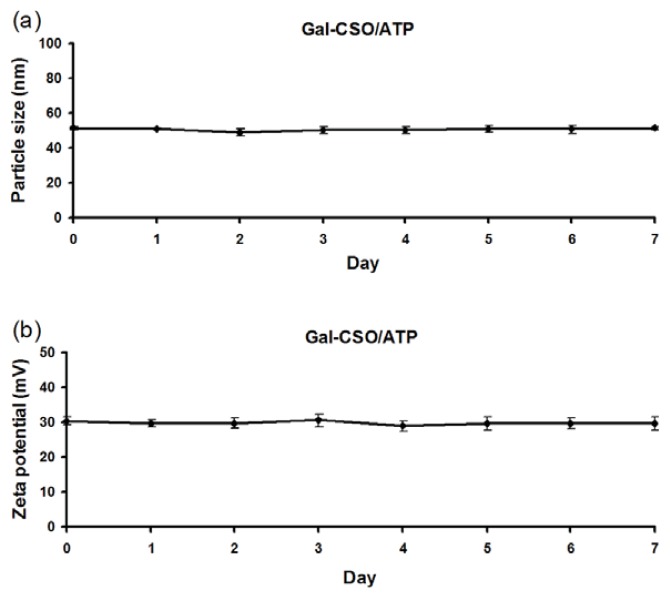
Stability of Gal-CSO/ATP nanoparticles. (**a**) The average particle size and (**b**) zeta potential of Gal-CSO/ATP remained stable for up to seven days after synthesis. Data represent the mean ± standard deviation (*n* = 3).

TEM images showed that the nanoparticles appeared spherical in shape (Figure 2). The physicochemical properties of CSO/ATP and Gal-CSO/ATP are summarized in Table 1. Zeta potentials of CSO/ATP and Gal-CSO/ATP were around +40 and +30 mV, respectively. Compared with CSO/ATP, the Gal-CSO/ATP had a larger size and lower zeta potential. The average particle size (Figure 3a) and zeta potential (Figure 3b) of Gal-CSO/ATP exhibited negligible changes when nanoparticles were incubated with pH 7.4 PBS up to seven days, indicating that Gal-CSO/ATP nanoparticles maintained their stability under physiological condition and might be suitable for *in vivo* application.

In this research, Gal-CSO showed great ability to form a complex with ATP and proper physicochemical properties for a drug delivery carrier. Particle size plays an important role in transferring drug to the cells, and we tried to obtain nanoparticles of a size below 200 nm in order to facilitate the uptake of the particles. The particle size for Gal-CSO/ATP nanoparticles was found to be relatively higher than the particle size of CSO/ATP nanoparticles, which could be due to substitution of some amino group of CSO by a bulky lactobionate moiety and higher drug entrapment. The zeta potential, which indicates the present repulsive force and is widely used to predict the long-term stability of the nanoparticles, was determined. The two types of nanoparticles exhibited positive zeta potential, which explained the cationic nature of the CSO and synthesized Gal-CSO. Compared with CSO/ATP, the average zeta potential of Gal-CSO/ATP complexes was lower and positive, because of the decreased number of surface positive charges after galactose modification of the CSO.

#### 2.2.2. ATP Loading and *in Vitro* ATP Release

**Figure 4:**
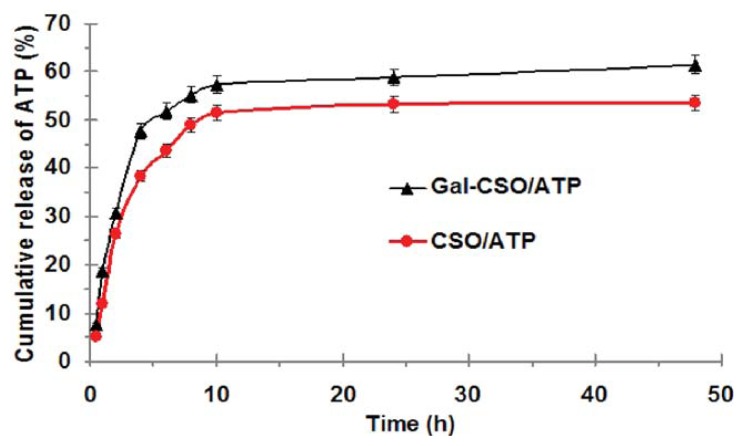
*In vitro* drug release profiles of Gal-CSO/ATP and CSO/ATP nanoparticles in PBS (pH 7.4) at 37 °C. Data represent the mean ± standard deviation (*n* = 3).

To further evaluate the preparation of the nanoparticles, encapsulation efficiency (EE) and drug loading (DL) were measured. As listed in Table 1, the DL and EE of Gal-CSO were higher than those of CSO. For tested nanoparticles, release of ATP revealed a biphasic pattern: an initial burst and a following slower and continued release. As shown in Figure 4, within the first 2 h, about 26.29% and 30.81% of ATP was released from CSO/ATP and Gal-CSO/ATP, respectively. After 48 h, the total amount of ATP released from CSO/ATP and Gal-CSO/ATP was 53.55% and 61.5%, respectively.

(1) EE (%, w/w)=[(Amount of ATP in nanoparticles)/(Total amount of ATP)]×100%

(2) $$\begin{array}{c} \text{DL }(\%,\;w/w)=[(\text{Amount of ATP}\;\text{in nanoparticles})/(\text{Amount of ATP in}\;\text{nanoparticles}+\\ \text{Weight of nanoparticles})]\times 100\% \end{array}$$

*In vitro* cumulative release rate profiles of ATP from Gal-CSO/ATP or CSO/ATP nanoparticles showed the initial phase of burst release, which is attributed to the drug located/adsorbed at the cross-linked surface of the nanoparticles. After the combination of CSO and galactose, part of the amino group in the CSO is combined with the carboxyl group in galactose, which leads to a reduction of positive charges of Gal-CSO, and hence, its combination with ATP is less compact than CSO. This observation could be explained by the fact that the cumulative release rate of Gal-CSO/ATP nanoparticles is relatively higher than that of CSO/ATP nanoparticles.

### 2.3. *In Vitro* Cellular Uptake

**Figure 5:**
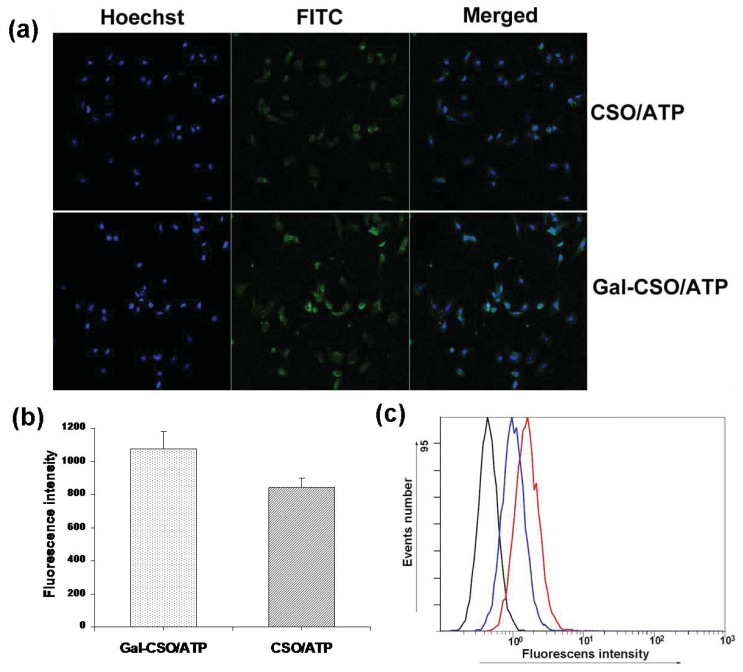
HepG2 cells were incubated with fluorescein isothiocyanate (FITC)-labeled Gal-CSO/ATP and CSO/ATP nanoparticles for 24 h, respectively. (**a**) Confocal laser scanning images; (**b**) the quantitative analysis based on the imaging in (**a**) by the software, “ImageJ”; and (**c**) quantitative cell uptake, analyzed by a flow-cytometer, of CSO/ATP-treated cells (blue lines) and Gal-CSO/ATP-treated cells (red lines).

Figure 5a shows the confocal laser scanning images of HepG2 cells after the cells were incubated with fluorescein isothiocyanate (FITC)-labeled Gal-CSO/ATP and CSO/ATP nanoparticles for 24 h, respectively. It was clear that the Gal-CSO/ATP nanoparticles could be uptaken by HepG2 cells, and the fluorescence intensity in Gal-CSO/ATP-treated cells was stronger than in CSO/ATP-treated cells (Figure 5b), which was confirmed quantitatively by the software, “ImageJ” (National Institutes of Health, Bethesda, MD, USA), suggesting that the uptake amount was relatively higher. These findings were in accordance with the results of flow cytometry in Figure 5c.

### 2.4. *In Vitro* Cytotoxicity

**Figure 6:**
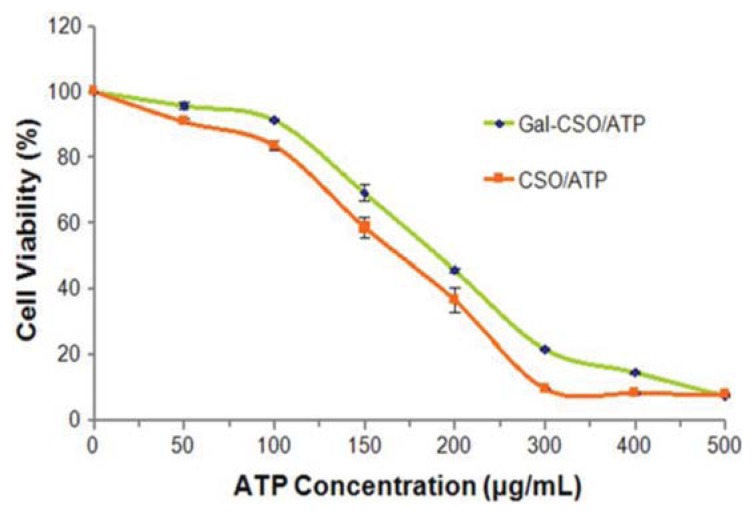
Cell viability % of HepG2 cells after incubation with Gal-CSO/ATP and CSO/ATP for 48 h, respectively. Cytotoxicity was evaluated by the methyl tetrazolium (MTT) assay. Data represent the mean ± standard deviation (*n* = 3).

*In vitro* cytotoxicity results at different concentrations of ATP are shown in Figure 6. The half maximal inhibitory concentration (IC_50_) values within 48 h could be calculated from the dose-responsive viability curves, which were 154.8 and 194.9 μg/mL for CSO/ATP and Gal-CSO/ATP in HepG2 cells, respectively. In general, the two types of nanoparticles showed low toxicity in HepG2 cells. The results further demonstrated that the nanoparticles we synthesized were biocompatible and safe.

(3) %Cell Viability=Mean experimental absorbance/Mean control absorbance×100%

Cell viability is a significant parameter to be evaluated in order to determine any cytotoxicity of biomaterials in *in vitro* settings. The predictive value of *in vitro* cytotoxicity tests is based on the concept that toxic chemicals affect the basic functions of cells, and such functions are common to all cells; hence, the toxicity can be measured by assessing cellular damage. Methyl tetrazolium (MTT) assay, which contains the reagent, 3-(4,5-dimethylthialzol-2-yl)-2.5-diphenyl tetrazolium bromide (MTT) prepared in deionized water, is one of the methods commonly used for this purpose. In this study, MTT assay is carried out to determine the cell viability of cells in response to the concentration of the two types of nanoparticles. The % viability of Gal-CSO/ATP nanoparticles was compared with CSO/ATP nanoparticles, and the 48 h exposure was chosen, as the cells would be within an exponential growth phase in this period, meaning that any toxicity, due to inhibition of proliferation, would be clearly visible in the MTT assay. The results showed that significant cytotoxicity was not observed for two types of nanoparticles in the HepG2 cell line. Therefore, it is expected that the Gal-CSO/ATP nanoparticles will have a great potential for safe hepatocyte-targeting drug delivery applications.

## 3. Experimental Section

CSO (*M*_w_ = 4600 Da; degree of acetylation = 5%) was obtained by enzymatic hydrolysis of chitosan in our lab [36]. LA was obtained from Acros Organics (Morris Plains, NJ, USA). EDC and tetramethylethylenediamine (TEMED) were purchased from Aldrich Chemical Company (Milwaukie, WI, USA). ATP was purchased from Hangzhou Meiya Biotechnical Co. Ltd. (Hangzhou, China). All other reagents and solvents used were of analytical reagent grade. Water used in this study was deionized. Human hepatocellular carcinoma cell line, HepG2, was purchased from the Institute of Biochemistry and Cell Biology (Shanghai, China) and cultured in Dulbecco’s Modified Eagle Medium (DMEM) (Gibco, NY, USA) containing 10% fetal bovine serum (FBS) at 37 °C in a humidified incubator with 5% CO_2_ and 95% relative humidity.

The Gal-CSO was synthesized by directly coupling LA with CSO according to a similar method previously described by Chung *et al.* [29]. LA was coupled with CSO using EDC as the coupling agent. Briefly, 106.3 mg LA dissolved in 0.5 mL of TEMED/HCl buffer solution (pH 4.7) were activated with EDC (556.7 mg). Subsequently, 0.5 g CSO was added into the solution at an equivalent molar ratio to LA. The reaction was performed at 80 °C for about 5 h to let LA conjugate onto CSO molecules. The solution was dialyzed against deionized water for 24 h at room temperature, followed by lyophilization, and the Gal-CSO was received.

Nanoparticles were prepared by the similar method previously described [24]. Briefly, 0.01 g Gal-CSO or CSO and 0.01 g ATP were first dissolved in 10 mL deionized water, respectively, and the mixture was stirred for 10 min by magnetic stirrer (400 rpm). Subsequently, ATP solution was dropwise mingled with the stirred Gal-CSO or CSO solution. When the transparency of the solution decreased accompanying an apparent Tyndall effect, this meant that the nanoparticles were obtained. Finally, the nanoparticles were prepared using the optimized parameters and characterized.

The particle size and surface charge of the prepared nanoparticles were assessed by a Zeta-sizer (3000HS, Malvern Instruments Ltd., UK) at a temperature of 25 °C. The samples of nanoparticles were prepared after the nanoparticles’ dispersion washed thrice with petroleum benzene by the help of centrifugation and re-dispersed in Deionization (DI) water.

The morphological examination of CSO/ATP and Gal-CSO/ATP was conducted by TEM (TECNAI 10, PHILIPS, Dutch) and measured by granule diameter. Appropriate amounts of samples were diluted with water and placed on copper grids covered with nitrocellulose, which were air-dried at room temperature and negative stained by 1% (*w*/*v*) phosphotungstic acid prior to the observation.

The ATP concentration was determined by measuring UV absorbance at 259 nm. The calibration curve of UV absorbance against ATP concentration was obtained using ATP PBS solution (pH 7.4). The UV absorbance of PBS was used as a blank. The good linear correlation was obtained in the range of 0.005–0.035 mg/mL. The regression equation was: *y* = 0.0414*x* − 0.0005 (*R*^2^ = 0.9999). The EE and DL were calculated from the ATP concentration in the water phase (PBS) during the separation process of nanoparticles and the charged amount of ATP.

In order to simulate the environment of blood and the internal environment of tumor cells, PBS (pH 7.4) was used as the dissolution medium for the *in vitro* ATP release tests from the nanoparticles. After the nanoparticles’ dispersion was washed thrice with PBS (pH 7.4) solution, the nanoparticles were re-dispersed in 25 mL PBS (pH 7.4) solution, and the dispersion was then placed in an incubator shaker (SHELLAB1227-2E, SHELLAB, Cornelius, OR, USA), which was maintained at 37 °C and shaken horizontally at 60 rpm. One milliliter of the dispersion was withdrawn from the system at predetermined time intervals, and the dispersion was centrifuged (21,000 rpm) for 10 min, following filtration with a 100 nm filter. The ATP concentration in the filtrate was assayed by ultraviolet spectrophotometry as described above. The accumulative release percentage was calculated from the established standard curve.

For investigating cellular uptake, HepG2 cells were seeded onto 10 mm coverslips in 24-well plates (Nalge Nune Interational, Naperville, IL, USA) at 5 × 10^4^ cells per well and cultured for 24 h. Cells were then incubated with FITC-labeled nanoparticles dispersion in growth medium for another 24 h. Cell nuclei were stained with Hoechst for 30 min. Following the incubation, cells were washed thrice with PBS and, then, fixed with fresh 4% paraformaldehyde at 4 °C for 20 min. The coverslips were observed by a confocal laser scanning microscope (LSM-510 META, ZEISS, Heidelberg, Germany). Parameters of fluorescence intensity for image optimization of fluorescently-labeled cells were measured using the Java image processing software, “ImageJ”. For the quantitative analysis of cell uptake, cells were treated with trypsin after 24-h incubation with the two kinds of nanoparticles, respectively, and, then, re-suspended in PBS. The intensity of cellular fluorescence was evaluated by a flow-cytometer (FC500MCL, Beckman Coulter, Fullerton, CA, USA).

In order to investigate the cytotoxicity of the ATP loaded nanoparticles, the methyl tetrazolium (MTT) assay was carried out according to the method described previously [37]. HepG2 cells were seeded in 24-well plates at a density of 5 × 10^4^ cells per well and cultured for 24 h. The cells were then incubated with ATP loaded nanoparticles at the ATP-equivalent dose of 50, 100, 150, 200, 300, 400 and 500 μg/mL for 48 h, respectively. After incubation, 20 μL of 5 mg/mL MTT solution in PBS (pH 7.4) were added to each well, and the plate was incubated for another 1 h; the medium including free non-adhered cells was thoroughly washed with PBS (pH 7.4) three times. The percentage cell viability was determined by measuring the absorbance at 570 nm using an ELISA plate reader (Bio-Rad, Microplate Reader 3550, Hercules, CA, USA). The cell viability was calculated as the percentage of MTT absorbance as follows: All experimental data were expressed as the mean ± SD. Statistical significance was determined using the Student’s *t*-test between two groups. The differences were judged to be significant at *p* < 0.05.

## 4. Conclusions

In this study, we demonstrated that Gal-CSO, as a derivative of CSO, has the ability to form nanoparticles when loading with ATP. It showed suitable physicochemical properties for a drug delivery system. Cytotoxicity of the nanoparticles was investigated with the MTT assay, which allows the quantification of the cell metabolic activity. Furthermore and most importantly, the *in vitro* analysis using HepG2 cells suggested that the Gal-CSO/ATP nanoparticles have a more specific uptake capacity when compared with CSO/ATP nanoparticles. Meanwhile, this study enabled us to understand the interactions of Gal-CSO/ATP nanoparticles with HepG2 cells *in vitro* before their use *in vivo*. Furthermore, *in vivo* or clinical experiments should also be performed to test the system as a novel hepatocyte-targeting drug delivery vector for its safety, novelty, as well as applicability for medical purposes, and this work is ongoing in our lab. The presented results indicated that the Gal-CSO/ATP nanoparticles might be very attractive to be used as a drug delivery carrier for hepatocyte targeting, thus warranting further *in vivo* or clinical investigations.

## Figures and Tables

**Table 1:** Physicochemical characteristics of ATP loaded nanoparticles. Data represent the mean ± standard deviation (*n* = 3). DL, drug loading; EE, encapsulation efficiency.

Sample	Particle size (nm)	Zeta potential (mV)	DL (%)	EE (%)
CSO/ATP	37.73 ± 1.27	43.58 ± 3.21	23.91 ± 0.1	78.58 ± 0.6
Gal-CSO/ATP	51.03 ± 3.26	30.50 ± 1.25	26.25 ± 0.1	88.98 ± 0.5
